# Knowledge and Attitude towards Cervical Cancer and Human Papillomavirus among Pharmacists in Japan

**DOI:** 10.31557/APJCP.2021.22.7.2259

**Published:** 2021-07

**Authors:** Ai Sasaki, Yuki Nakao, Taku Obara, Shinya Abe, Hiroshi Yamaguchi, Shoko Yoshimachi, Teruaki Goto

**Affiliations:** 1 *Tsuruha Pharmacies Co., Ltd. Sapporo, Hokkaido, Japan. *; 2 *Division of Preventive Medicine and Epidemiology, Tohoku University Tohoku Medical Megabank Organization, Sendai, Miyagi, Japan. *; 3 *Department of Molecular and Epidemiology, Tohoku University Graduate School of Medicine, Sendai, Miyagi, Japan. *; 4 *Department of Pharmaceutical Sciences, Tohoku University Hospital, Sendai, Miyagi, Japan. *; 5 *Tsuruha Holdings Inc, Sapporo, Hokkaido, Japan. *

**Keywords:** cervical cancer, human papillomavirus-, Japan, pharmacists, papillomavirus vaccines

## Abstract

**Objective:**

The objective of this study was to assess the knowledge and attitude towards cervical cancer and human papillomavirus (HPV) among pharmacists in Japan.

**Methods:**

Questionnaires were disseminated to 788 pharmacists employed by the Tsuruha Holdings Inc. A total of 617 pharmacists responded, generating a response rate of 78.3%.

**Result:**

Of the 362 females and 255 males, vaccination rates were 14.4% and 0.8%, respectively. In terms of cervical cytology, 35.1% of females received it once every two years, and 26.2% received it irregularly. As for HPV testing, 12.2% of females received it once every two years, and 16.6% received it irregularly. The rate of “school curriculum” as an information source was significantly higher among younger pharmacists; while “internet”, “media”, “training seminar for pharmacist”, “advertisement in medical institution”, “internal manual”, and “others” were significantly higher among older pharmacists. The proportion of pharmacists with knowledge on general questions, except for those about HPV testing, was significantly higher among females than males. The vaccination rates of younger pharmacists were significantly higher than those of older pharmacists. The screening rates of cervical cytology were significantly higher among older than younger pharmacists, and also among those with at least 10 years of experience than those with less. There were no differences in the screening rates of HPV testing according to age or pharmacist experience.

**Conclusion:**

The proportion of pharmacists with knowledge about cervical cancer and HPV significantly varied depending on sex, age, and experience as a pharmacist. This study suggested that spreading the knowledge about cervical cancer and HPV might be effective for increasing the rates of cervical cancer screening.

## Introduction

Cervical cancer is one of the most common cancers of the female genital system globally. In 2018, approximately 570,000 women were diagnosed with cervical cancer, and 311,000 died from it (GLOBOCAN, 2019). Cervical cancer is caused by sexually acquired infections with certain types of human papillomavirus (HPV), particularly, HPV types 16 and 18 which cause 70% of cervical cancers and precancerous cervical lesions (WHO, 2020). Comprehensive cervical cancer control includes HPV vaccination as primary prevention, screening and treatment of pre-cancerous lesions as secondary prevention, diagnosis and treatment of invasive cervical cancer tertiary prevention as tertiary prevention and palliative care (WHO, 2020). The significance of primary and secondary prevention of HPV-related diseases has become a worldwide concern. In 2018, the International Papillomavirus Society stated that in order to eliminate cervical cancer as a public health problem, high coverage HPV vaccination for adolescents and high coverage cervical cancer screening, should be done in combination with appropriate treatment for all women (Garland et al., 2018).

In April 2013, the Japanese Ministry of Health, Labour and Welfare (MHLW) implemented the national HPV vaccine program which provided free immunization for girls aged 12-16 years (Simms et al., 2020). However, in June 2013, the program was suspended after unconfirmed reports of adverse events following vaccination appeared in the media (Gilmour et al., 2013). Since then, completion rates of the three-dose HPV series fell to less than 1% in 2013 from 68.4-74.0% in 2007-2011 (Hanley et al., 2015), despite the vaccine still being part of the national immunization program and available for free. Japan has since become a country with strong HPV vaccine hesitancy due to widespread public suspicion of adverse events. As for cervical cancer screening in Japan, the uptake rates within two years approximated at 42.4% (Japanese MHLW, 2016), this reflected the failure of reaching the government target of 50%, even though screening is provided at a subsidized price. Prevention strategies in Japan are insufficient as compared with other countries which achieve high vaccination and screening coverage such as Australia (Hall et al., 2019) and many other desirable countries (Bruni et al., 2016; Gakidou et al., 2008; OECD, 2013).

WHO reported that community pharmacists are the most accessible health professionals to the public and are cornerstones of primary health care (WHO, 2019). Community pharmacists play a key role in health promotion campaigns, locally and nationally, on a wide range of health-related topics. In 2016, the Japanese MHLW introduced the Health Support Pharmacy (HSP) system to promote awareness of public health through community pharmacy services, including dementia support, nutritional guidance, and health consultation, among others. However, it was reported that service quality differed across pharmacies, and adequate resources and professional expertise are required to improve the quality of pharmacy-based public health services (Sato et al., 2020). Pharmacists have a role in spreading appropriate knowledge and in encouraging preventive behavior of cervical cancer. Although several investigations have clarified the state of knowledge and attitude towards HPV and cervical cancer among the general population in Japan (Miyagi et al., 2014; Sukegawa et al., 2015; Suzuki et al., 2019), the knowledge and attitude among pharmacists and the relationship between them are unknown. The objective of this study was therefore to assess the knowledge and attitude towards cervical cancer and HPV and the relationship between them among pharmacists in Japan.

## Materials and Methods

Pharmacists employed by the Tsuruha Holdings Inc., a Japanese drugstore chain, were surveyed. A self-administered questionnaire was used to assess their knowledge about cervical cancer, HPV, and cervical cancer screening, as well as their experiences of HPV vaccination and cervical cancer screening (Suppl 1). Tsuruha Holdings Inc. headquarters disseminated these questionnaires to 788 pharmacists, who were asked to submit them through a web system between June 4-24, 2019. A total of 617 pharmacists responded, giving a response rate of 78.3%. The results were analyzed by stratification according to sex, age (<30 years, 30-39 years, 40-49 years, and ≥50 years), and experience as a pharmacist (<10 years and ≥10 years). The participants who responded “I understand” to the question regarding their understanding of cervical cancer and HPV were classified into the “Yes” group, while those who answered “I have heard, but don’t understand” or “I don’t know” were classified into the “No” group. Participants in the “Yes” group were considered to have knowledge about the topic of each question on cervical cancer and HPV. “HPV testing” means HPV testing alone, not including co-testing with cervical cytology. We compared the sources of information according to age. We also compared the attitude towards HPV vaccination and cervical cancer screening including cervical cytology and HPV testing in the same manner. Statistical analyses were performed by the Fisher’s exact probability test and the Cochran-Armitage trend test using the SAS package (version 9.4; SAS Institute Inc., Cary, NC, USA).

## Results

Of the 617 pharmacists, 58.8% were female, 70.5% were <40 years, and 55.7% had <10 years of pharmacist experience. Of the 362 females and 255 males, vaccination rates were 14.4% and 0.8%, respectively. In terms of cervical cytology, 35.1% of females received it once every two years, and 26.2% received it irregularly. As for HPV testing, 12.2% of females received it once every two years, and 16.6% received it irregularly.

*Source of information on cervical cancer according to age*

**Figure 1:**
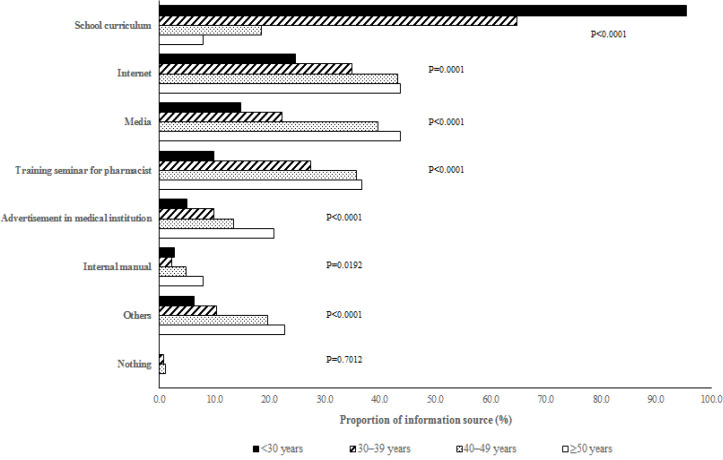
Source of Information on Cervical Cancer According to Age. P-values were expressed the results from the Cochran-Armitage trend test among age groups

The source of information on cervical cancer were different across age groups (Figure 1). The rate of “school curriculum” as an information source was significantly higher among younger pharmacists; while “internet”, “media”, “training seminar for pharmacist”, “advertisement in medical institution”, “internal manual”, and “others” were significantly higher among older pharmacists.

*Knowledge related to cervical cancer and HPV*

The proportion of pharmacists with knowledge on general questions, except for those about HPV testing, was significantly higher among females than males (Table 1). Between the age groups, knowledge about the relationship between HPV and cervical cancer, the transmission route of HPV, and HPV testing were significantly higher among younger pharmacists; while knowledge about cervical cytology and the subsidization of cervical cancer screening were significantly higher among older pharmacists. In terms of pharmacist experience, knowledge about the relationship between HPV and cervical cancer, the transmission route of HPV, and the HPV testing were significantly higher among those who had worked as a pharmacist for less than 10 years, while knowledge about cervical cytology and the subsidization of cervical cancer screening were significantly higher among pharmacists with at least 10 years of experience.

*Attitude towards HPV vaccination according to characteristics among female pharmacists*

The vaccination rates of younger pharmacists were significantly higher than those of older pharmacists (Table 2). Vaccination rates were also significantly higher in pharmacists with less than 10 years of experience than those with at least 10 years. The proportions of female pharmacists who have received HPV vaccines were significantly higher among those with knowledge about the transmission route of HPV and HPV vaccines, as compared to those without such knowledge.

*Attitude towards cervical cytology among female pharmacists*

The proportion of female pharmacists who received cervical cytology, be it once every two years or irregularly, were significantly higher among older than younger pharmacists, and also among those with at least 10 years of experience than those with less (Table 3). Rate of cervical cytology was also significantly higher among pharmacists with knowledge about cervical cancer, HPV, cervical cytology, the differences between cervical cytology and HPV testing, and the subsidization of cervical cancer screening, compared to those without such knowledge. Lastly, cervical cytology rate was significantly higher among pharmacists without knowledge about HPV testing, compared to those with this knowledge.

*Attitude towards HPV testing among female pharmacists*

There were no differences in the screening rates of HPV testing (once every 2 years or irregularly) according to age or pharmacist experience (Table 4). HPV testing rate was significantly higher among pharmacists with knowledge about cervical cancer, HPV, the relationship between HPV and cervical cancer, HPV vaccines, HPV testing, the differences between cervical cytology and HPV testing, and the subsidization of cervical cancer screening, compared to those without such knowledge. HPV testing rate was also significantly higher among the pharmacists without knowledge about cervical cytology, compared to those with this knowledge.

**Table 1:** Knowledge Related to Cervical Cancer and HPV

		Sex			Age					Experience as pharmacist		
		Female n=362	Male n=254	P*	<30 years n=223	30-39 years n=211	40-49 years n=81	≥50 years n=101	P**	<10 years n=343	≥10 years n=273	P*
Do you know about cervical cancer which develops in uterine cervix?												
	Yes, %	71.3	60.6	0.006	66.4	63.5	64.2	77.2	0.111	65.3	68.9	0.389
Do you know about HPV with which most people experience infection?												
	Yes, %	68.8	50.4	<0.0001	65.9	57.8	56.8	61.4	0.288	62.4	59.7	0.507
Do you know that persistent infection with HPV can lead to cervical cancer?												
	Yes, %	77.4	69.7	0.032	82.5	70.6	71.6	65.4	0.001	79.3	67.8	0.002
Do you know that HPV can be transmitted by sexual intercourse?												
	Yes, %	84.3	77.2	0.026	90.1	78.7	75.3	72.3	<0.0001	86.0	75.5	0.001
Do you know about HPV vaccines?												
	Yes, %	69.9	54.3	<0.0001	72.7	54.5	58.0	66.3	0.135	66.8	59.3	0.064
Do you know about HPV testing?												
	Yes, %	11.1	24.8	<0.0001	22.0	15.6	12.4	10.9	0.006	20.4	12.1	0.007
Do you know about cervical cytology?												
	Yes, %	35.4	19.3	<0.0001	21.1	31.8	33.3	35.6	0.004	23.0	35.9	0.001
Do you know differences of the cervical cytology and the HPV testing?												
	Yes, %	30.4	18.5	0.001	26.0	20.9	19.8	38.6	0.073	24.5	26.7	0.577
Do you know that you can receive cervical cancer tests at a subsidized price by companies or governments?												
	Yes, %	69.6	34.7	<0.0001	50.2	55.5	59.3	62.4	0.028	51.3	60.1	0.034

HPV, human papillomavirus; *, Fisher’s exact test; **, Cochran-Armitage trend test.

**Table 2:** Attitude towards HPV Vaccination among Female Pharmacists

	HPV vaccination rate, %	P*
Age		
<30 years	29.2	<0.0001**
30-39 years	9.5	
40-49 years	3.8	
≥50 years	0.0	
Experience as pharmacist		
<10 years	22.4	<0.0001
≥10 years	4.4	
Do you know about cervical cancer which develops in uterine cervix?		
No	19.2	0.100
Yes	12.4	
Do you know about HPV with which most people experience infection?		
No	14.2	1.000
Yes	14.5	
Do you know that persistent infection with HPV can lead to cervical cancer?		
No	9.8	0.212
Yes	15.7	
Do you know that HPV can be transmitted by sexual intercourse?		
No	5.3	0.038
Yes	16.1	
Do you know about HPV vaccines?		
No	7.3	0.014
Yes	17.4	
Do you know about cervical cytology?		
No	13.0	0.054
Yes	25.0	
Do you know about HPV testing?		
No	15.0	0.755
Yes	13.3	
Do you know differences of the cervical cytology and the HPV testing?		
No	15.9	0.256
Yes	10.9	
Do you know that you can receive cervical cancer tests at a subsidized price by companies or governments?		
No	19.1	0.104
Yes	12.3	

HPV, human papillomavirus; *, Fisher’s exact test; **, Cochran-Armitage trend test.

**Table 3:** Attitude towards Cervical Cytology among Female Pharmacists

	Once every two years, %	P*	"Once every two years or irregular, %"	P*
Age				
<30 years	21.2	<.0001**	42.3	<0.0001**
30-39 years	34.3		70.5	
40-49 years	49.1		79.3	
≥50 years	53.7		71.6	
Experience as pharmacist				
<10 years	23.4	<0.0001	48.3	<0.0001
≥ 10 years	49.7		77.6	
Do you know about cervical cancer which develops in uterine cervix?				
No	21.2	0.0004	45.2	<0.0001
Yes	40.7		67.8	
Do you know about HPV with which most people experience infection?				
No	25.7	0.013	51.3	0.010
Yes	39.4		65.9	
Do you know that persistent infection with HPV can lead to cervical cancer?				
No	29.3	0.237	53.7	0.122
Yes	36.8		63.6	
Do you know that HPV can be transmitted by sexual intercourse?				
No	26.3	0.173	57.9	0.558
Yes	36.7		62.0	
Do you know about HPV vaccines?				
No	27.5	0.055	61.5	1.000
Yes	38.3		61.3	
Do you know about HPV testing?				
No	38.5	0.0001	65.8	<0.0001
Yes	7.5		25.0	
Do you know about cervical cytology?				
No	31.2	0.036	53.4	<0.0001
Yes	42.2		75.8	
Do you know differences of the cervical cytology and the HPV testing?				
No	30.2	0.004	57.9	0.047
Yes	46.4		69.1	
Do you know that you can receive cervical cancer tests at a subsidized price by companies or governments?				
No	15.5	<0.0001	42.7	<0.0001
Yes	43.7		69.4	

HPV, human papillomavirus; *, Fisher’s exact test; **, Cochran-Armitage trend test.

**Table 4:** Attitude towards HPV Testing among Female Pharmacists

	Once every two years, %	P*	"Once every two years or irregular, %"	P*
Age				
<30 years	10.2	0.319**	28.5	0.806**
30-39 years	12.4		30.5	
40-49 years	13.2		28.3	
≥50 years	14.9		26.9	
Experience as pharmacist				
<10 years	10.0	0.195	29.4	0.816
≥ 10 years	14.9		28.0	
Do you know about cervical cancer which develops in uterine cervix?				
No	5.8	0.020	17.3	0.002
Yes	14.7		33.3	
Do you know about HPV with which most people experience infection?				
No	8.0	0.119	15.0	<0.0001
Yes	14.1		34.9	
Do you know that persistent infection with HPV can lead to cervical cancer?				
No	7.3	0.177	18.3	0.018
Yes	13.6		31.8	
Do you know that HPV can be transmitted by sexual intercourse?				
No	3.5	0.027	19.3	0.110
Yes	13.8		30.5	
Do you know about HPV vaccines?				
No	4.6	0.003	18.4	0.005
Yes	15.4		33.2	
Do you know about HPV testing?				
No	12.7	0.447	29.5	0.459
Yes	7.5		22.5	
Do you know about cervical cytology?				
No	14.5	0.066	33.8	0.005
Yes	7.8		19.5	
Do you know differences of the cervical cytology and the HPV testing?				
No	8.7	0.005	23.0	0.0004
Yes	20.0		41.8	
Do you know that you can receive cervical cancer tests at a subsidized price by companies or governments?				
No	4.6	0.003	15.5	0.0002
Yes	15.5		34.5	

HPV, human papillomavirus; *, Fisher’s exact test; **, Cochran-Armitage trend test.

## Discussion

Many reports have revealed a positive correlation between knowledge and vaccination coverage (Oz et al., 2018; Navalpakam et al., 2019; Liu et al., 2020); however, few reports have examined the correlation between knowledge and screening coverage. Worldwide simulations have shown that the proper combination of HPV vaccine and screening is important (Garland et al., 2018). We therefore investigated the statuses of both HPV vaccine and cervical cancer screening, and to our knowledge, this is the first report that investigated the correlations between knowledge and these statuses.

The information source of cervical cancer were greatly different across age groups. Younger pharmacists tended to be informed by school curriculums, while older pharmacists were mainly informed by the internet, the media, and training seminars. Younger pharmacists had more opportunities to learn about cervical cancers in school because the relevance between cervical cancer and HPV became apparent in the 1980s and early 1990s (Lowy et al., 2008), hence contributing to their rich knowledge about cervical cancer and HPV. These results suggested that educating cervical cancer in school is exceptionally effective. For pharmacists aged ≥40 years, edification through the internet, media, and training seminars seemed to be effective.

In Japan, cervical cytology is the standard approach for cervical cancer screening and it is covered by insurance. HPV testing, in contrast, is only covered by insurance when patient is diagnosed with atypical squamous cells of undetermined significance (ASC-US) by cervical cytology. Unlike overseas, HPV testing is not widespread in Japan and only a few individuals have experience undergoing the test. However, in this study, 12.2% of females received HPV testing once every two years, and 16.6% received it irregularly. The screening rates of HPV testing in this study seem to be high. Some pharmacies to which the participants belong provides service of HPV testing on self-samples to the customer. Purchasing the service themselves and receiving HPV testing might cause an increase in the screening rates.

Of the 362 female pharmacists, 35.1% received cervical cytology once every two years. In contrast, some westernized countries have reported screening rates as high as 80% (OECD, 2013). Only 21.2% of pharmacists aged <30 years received cervical cytology once every two years, despite screening being recommended to begin at age 20 in Japan. This tendency towards lower screening rates among younger pharmacists is consistent with the Comprehensive Survey of Living Conditions of the general population (Japanese MHLW, 2016). Our survey showed that the HPV vaccination rate of pharmacists aged <30 years was remarkably high at 29.2%. This result seemed to be caused by the fact that 20-25 year-old females during the investigation period were subjects of the national HPV vaccine program. The HPV vaccination rate was significantly higher only with the knowledge about HPV vaccines and the transmission route of HPV, whereas no differences were observed in other overall knowledge about cervical cancer and HPV. These results indicated that vaccination status is more influenced by whether or not the subjects were part of the HPV vaccine program, rather than the presence or absence of knowledge. Suspending active recommendation for HPV vaccination, rather than the hindrance of information dissemination, hence imposes a greater impact towards vaccination rates. The dissemination of knowledge is obviously important; however, government recommendations and the national HPV vaccine program are more effective for increasing vaccination. The Japan Society of Obstetrics and Genecology considered that HPV vaccination is necessary from a scientific view and has issued a statement strongly urging the government to resume recommendations for the HPV vaccination (Japan Society of Obstetrics and Genecology, 2015). On the other hand, the proportion of pharmacists who underwent cervical cytology and HPV tests (once every two years or irregularly) was significantly higher among those with knowledge about cervical cancer and HPV. This indicated that the spreading of adequate knowledge is effective in promoting cervical cancer screening. In fact, a previous study showed that low perceived susceptibility to the disease and the lack of knowledge are the possible barriers of cervical cancer screening (Oshima and Maezawa, 2013).

According to a previous survey by Suzuki et al., (2019) in Japan, in the laypersons’ group, females had a significantly higher level of knowledge about cervical cancer and HPV than males; whereas in the medical profession group, there were no differences in level of knowledge between genders. However, in our survey, the proportion of pharmacists with knowledge about cervical cancer and HPV was significantly higher among females than males. This inconsistency seemed to be caused by the differences in job category of the participants and in the method of assessing knowledge. In the survey by Suzuki et al., (2019), the medical profession group included medical school students, nursing school students, other medical students, medical doctors, nurses, public health nurses, midwifes, pharmacists, medical laboratory technicians and other medical professionals. They conducted the survey in 21 locations, including public spaces, classrooms, company offices, university festivals, citizens’ open forums and an academic meeting, and assessed the knowledge level of cervical cancer and HPV by correct answer rates for 11 questions. It was stated that one of the limitations of their study was selection bias, in that the participants might have had more interest in health promotion, cervical cancer or HPV. The HPV vaccine is known and described as a “cervical cancer preventive vaccine”, and little is known about the prevention of other HPV-related cancers in Japan. The importance of HPV vaccination in boys have attracted global attention, because high-risk HPV can also cause other cancers such as vaginal, vulvar, anal, penile, oropharyngeal, and oral cancers (Lowy et al., 2008). In Australia, the vaccination rate of 15-year-old boys in 2016 was reported to be 72.9% (Hall et al., 2019). Therefore, the risk of HPV-related cancers should not be disregarded in males.

This study indicated that the pharmacists’ knowledge varied widely depending on their characteristics such as sex, age, and experience as a pharmacist. Ideally, all pharmacists should have a high level of knowledge level regardless of their characteristics. Therefore, the knowledge gap should be filled by adequate resources. Previous studies have reported a positive influence of medical professionals on the acceptability of the HPV vaccination (Gamble et al., 2010; Khan et al., 2016). Pharmacists with high knowledge and a sense of mission are essential in assisting the understanding of cervical cancer and HPV in the general population, and also in promoting HPV vaccination and cervical cancer screening. Our findings advocate the improvement of recommendation behaviors in community pharmacists and the spreading of knowledge for preventing and screening cervical cancer.

In conclusion, our survey revealed that pharmacists’ knowledge and attitude towards cervical cancer and HPV significantly varied depending on sex, age, and experience as a pharmacist. This study suggested that the spreading of knowledge about cervical cancer and HPV might be effective in increasing not only the rate of HPV vaccination but also the rate of cervical cancer screening. 

## Author Contribution Statement

All authors contributed to this scientific work and approved the final version of the manuscript. AS and designed this study and wrote the manuscript. YN, KF, and MH were deeply involved in the design of the study. TO performed the data analyses and co-wrote the manuscript. SA supervised the data analyses and co-wrote the manuscript. SY and TG critically revised the manuscript.
